# Characterization of the (Poly)Phenolic Fraction of Fig Peel: Comparison among Twelve Cultivars Harvested in Tuscany

**DOI:** 10.3390/plants11223073

**Published:** 2022-11-13

**Authors:** Luca Calani, Letizia Bresciani, Margherita Rodolfi, Daniele Del Rio, Raffaella Petruccelli, Cecilia Faraloni, Tommaso Ganino

**Affiliations:** 1 Department of Food and Drug, University of Parma, Parco Area delle Scienze 27/A, 43124 Parma, Italy; 2 Institute of BioEconomy (IBE-CNR), via Madonna del Piano 10, 50019 Sesto Fiorentino, Italy

**Keywords:** *Ficus carica* L., fig peel, liquid chromatography, mass spectrometry, rutin, 5-caffeoylquinic acid, anthocyanins

## Abstract

(1) Background: The fig tree (*Ficus carica* L.) is widely cultivated in the Mediterranean area and it produces fruits largely consumed in the Mediterranean diet. Previous studies have shown that this fruit represents a rich source of (poly)phenols, which are mainly located in the peel rather than the pulp. In our study, fig peel derived from twelve different cultivars located in Tuscany was assessed for its (poly)phenol profile. (2) Methods: The (poly)phenol characterization was performed through ultra-high performance liquid chromatography coupled to multiple-stage mass spectrometry. (3) Results: Twenty-eight (poly)phenolic compounds were quantified in the investigated fig peel. It was possible to observe an interesting variability in the (poly)phenol content among the twelve cultivars of fig peel. Rutin and 5-caffeoylquinic acid were the main compounds in the greenish fig peel, while cyanidin-3-*O*-rutinoside was the main component in the dark-violet fig peel. (4) Conclusions: fig peel could be used as a (poly)phenol-rich ingredient in several food products to increase the bioactive compound content of foods. Moreover, dark-violet peel could be considered potentially suitable as a natural food colorant.

## 1. Introduction

The fig *(Ficus carica* L.) is one of the earliest cultivated fruit trees and an important crop worldwide for both dry and fresh consumption. Most of the world’s fig production is located in the Mediterranean countries. The fig has an important role in the Mediterranean diet, and is associated with a healthy lifestyle, longevity and a lower risk of developing several chronic-degenerative diseases [1,2]. The high consumption of fruit and vegetables, which is the basis of the Mediterranean diet, provides a wide range of beneficial compounds, including vitamins, dietary fibers and phytochemicals, including (poly)phenols. This class of non-nutrient bioactive compounds has been largely associated with protective effects against chronic diseases [3,4,5,6].

The fig represents a typical Mediterranean fruit, widely used in different Italian dishes and by the confectionary industry. Although fig fruits are mainly consumed after peeling, the dietary consumption of whole ripe fruits rather than peeled fruits should be encouraged as a possible dietary strategy to increase daily (poly)phenol intake; the use of whole ripe fig fruits is applied in the preparation of several dishes regularly consumed in Mediterranean countries. However, the partial use of the peeled fruit by the confectionary industry leads to the generation of fig peel, a by-product that could represent an interesting source of anthocyanins and colorless (poly)phenols. Indeed, previous studies on fig-derived (poly)phenols discovered that these compounds are mainly located in the skin rather than the pulp [7,8,9,10,11,12,13], thus making the skin potentially reusable by the food industry to develop functional foods with increased (poly)phenol content, as recently proposed for fig seeds [14].

Therefore, the aim of the present study was to evaluate the (poly)phenolic profile of the peel of twelve cultivars of figs harvested in Tuscany as a potential (poly)phenol-rich ingredient for food production.

## 2. Results and Discussion

Preliminary untargeted analysis allowed the detection and the identification of the main (poly)phenols that characterize fig peel (Supplemental Table S1 and Supplemental Figures S1–S3). The concentration of the main (poly)phenolic compounds detected in the peel of the investigated fig cultivars is reported in Table 1. In detail, 28 (poly)phenolic compounds were quantified in the fig peel of twelve cultivars located in Tuscany. The (poly)phenol characterization reported in the present work mainly focused on fig skins because fig-derived (poly)phenols have been demonstrated to be mostly located in peel rather than pulp [7,8,9,10,11,12]. Quercetin-3-*O*-rutinoside (also known as rutin) was by far the most abundant flavonoid in the greenish fig peel although significant differences emerged among the twelve cultivars. Rutin reached a content close to 200 µg/g dry weight (DW) in the ‘Portogallo’ cultivar (F8), while the lowest rutin content was observed in the ‘Pecciolo bianco’ cultivar (F7), with values lower than 75 µg/g DW (Table 1). Besides rutin, the ‘Portogallo’ cultivar (F8) showed the highest content of the other flavonol glycosides, such as quercetin-*O*-hexoside, quercetin-*O*-acetylhexoside and kaempferol-*O*-rutinoside. The current study confirmed that flavonols, mainly rutin, are the most abundant flavonoids in greenish fig peel, as previously observed [10,11]. As expected, several cyanidin derivatives, mainly glycosides, were recovered only in four cultivars, namely ‘Brogiotto nero’ (F2), ‘Corbo’ (F3), ‘Portogallo’ (F8) and ‘Pecciolo nero’ (F9). As reported in Table 1, cyanidin-3-*O*-rutinoside was by far the most abundant cyanidin glycoside in all four cultivars, reaching the highest content in ‘Corbo’ (2610 µg/g dry weight). The same cultivar also showed the highest values of the other quantified cyanidin glycosides in comparison to ‘Brogiotto nero’ (F2), ‘Portogallo’ (F8) and ‘Pecciolo nero’ (F9). Besides cyanidin glycosides, ‘Corbo’ was the cultivar with the highest content of monomeric flavan-3-ols, namely (+)-catechin and (−)-epicatechin, as well as B-type procyanidin dimer, reaching an overall flavan-3-ol content close to 41 µg/g DW, about four-fold higher than the flavan-3-ol content of ‘Pecciolo nero’ (F9). Flavones in the investigated fig peel were recovered as *C*-glycosides, *O*-glycosides and *O*-glycosyl-*C*-glycosides, with several components significantly differing among the twelve investigated cultivars. It is interesting to note that apigenin-*O*-rutinoside was only recovered in ‘Brogiotto nero’ (F2), ‘Portogallo’ (F8) and ‘Corbo’ (F3), with the latter showing the highest content, equal to 27 µg/g DW.

Among phenolic acids, 5-caffeoylquinic acid was the most abundant compound, confirming previous findings on fig skin-derived phenolics [10,11,15]. As observed for flavonoids, a huge variability of 5-caffeoylquinic acid content occurred among the twelve sampled cultivars. This hydroxycinnamic acid reached a relevant content in the ‘Corbo’ (F3) and ‘Portogallo’ (F8) cultivars, with almost 131 and 111 µg/g DW, respectively. Besides 5-caffeoylquinic acid, the fig peel contained two additional caffeoylquinic acids, of which one was identified as 3-caffeoylquinic acid, mainly located in the ‘Portogallo’ (F8) cultivar, although it displayed a seven-fold lower content than 5-caffeoylquinic acid, considering the same cultivar.

The overall level of non-anthocyanin (poly)phenols ranged from 163 µg/g DW for ‘Dottato’ (F10) to 429 and 453 µg/g DW for the ‘Corbo’ (F3) and ‘Portogallo’ (F8) cultivars, respectively. Rutin and 5-caffeoylquinic acid accounted for more than 50% of the overall content of the non-anthocyanin (poly)phenols, pointing to fig peel as a good source of these phytochemicals, recently associated to health properties in humans [16,17,18,19]. The findings observed in the current study were consistent with a previous study where rutin and 5-caffeoylquinic acid were the most abundant non-anthocyanin (poly)phenols in the peel derived from Portuguese fig fruits [20]. Moreover, the ‘Brogiotto nero’ (F2), ‘Corbo’ (F3), ‘Portogallo’ (F8) and ‘Pecciolo nero’ (F10) cultivars contained relevant amounts of cyanidin glycosides, mainly cyanidin-3-*O*-rutinoside. The cultivar ‘Corbo’ (F3) showed a total anthocyanin content close to 2900 µg/g DW. Cyanidin-3-*O*-rutinoside contributed to a greater extent to the total anthocyanin content, as previously observed in figs harvested in Spain and Turkey [10,11,21]. Recent works have confirmed that cyanidin glycosides largely contributed on the overall anthocyanin content [22,23]. Thus, it is clear that dark-violet fig peel, especially from the ‘Corbo’ cultivar (F3), represent a good source of anthocyanins, bioactive compounds largely metabolized and absorbed [24], and associated to potentially beneficial properties [25,26].

Besides the quantification of the main (poly)phenolic compounds, sixteen minor (poly)phenols were further detected and reported in Table 2, with their occurrence in the fig peel of the investigated cultivars.

Although all the products were harvested in the same region, the results of the present study demonstrated a large variation in the (poly)phenol content of the fig peel depending on the cultivar. In accordance, Villamil-Galindo and colleagues demonstrated a significant variability of phenolic profile among different strawberry by-product cultivars [27]. Moreover, cultivar was reported also to be the main factor affecting the physicochemical and nutritional quality of kiwifruit and kiwi peel [28].

Being as the fig skin is a valuable source of (poly)phenolic compounds, as demonstrated in the present work, the consumption of the whole ripe fruit in a regular diet could be encouraged in the healthy population to improve the daily consumption of the potentially bioactive compounds, although its high sugar content must be taken into account in dietary recommendations.

From the other side, the industrial production of fig-based foodstuffs may lead to a high production of peel as a waste product. In the context of the circular economy applied to the agri-food chain [29,30], fig peel could be a valuable by-product that could be used as an innovative functional ingredient targeted to increase the (poly)phenol content of food products, especially by using the ‘Corbo’ (F3) and ‘Portogallo’ (F8) peel. Moreover, ‘Corbo’ (F3), being also rich in anthocyanins, could be a pigmented alternative useful to replace the synthetic colorants in several food products, besides its use in the development of (poly)phenol-enriched foodstuffs.

## 3. Materials and Methods

### 3.1. Chemicals

Rutin, (+)-catechin, 3-caffeoylquinic and 5-caffeoylquinic acids were purchased from Sigma-Aldrich (St. Louis, MO, USA), while vitexin, procyanidin B2, cyanidin-3-*O*-glucoside and cyanidin-3-*O*-rutinoside were from Extrasynthese (Genay Cedex, Genay, France). Water and acetonitrile were HPLC grade while formic acid was HPLC-MS grade and purchased from VWR (Milan, Italy). High-performance liquid chromotography-grade acetone was purchased from Carlo Erba Reagents (Milan, Italy).

### 3.2. Plant Material and Extraction of (Poly)Phenolic Fraction

The samples were derived from 12 different Italian fig cultivars collected in September 2013 from an experimental plot located in Carmignano (Tuscany, Italy). The overall information of all analyzed fig cultivars is reported in Table 3. The fig fruits were manually peeled, and peel was then freeze-dried (Heto Hetovac VR-1 and CT 110 Vacuum Concentrator, Heto Lab Equipment, Roskilde, Denmark) and subsequently homogenized using a mortar. All samples were then stored at −80% until (poly)phenol extraction. Dried fig (poly)phenols were extracted by accurately weighing 200 mg of freeze-dried powder in a plastic tube and by adding 5 mL of a solvent mixture containing acetone/water/formic acid, 80/19.5/0.5 (*v*/*v*/*v*) [10]. Tubes were vortexed for approximately 30 s, sonicated in an ultrasonic bath for 10 min, vortexed again for 30 s and finally centrifuged for 10 min at 2575 *g*. The supernatant was then evaporated through a centrifugal concentrator (SpeedVac SPD121P, Thermo Fisher Scientific Inc., San José, CA, USA) and the residual pellet was resuspended in 200 μL of 0.1% aqueous formic acid before uHPLC-MS^n^ analyses.

### 3.3. Analysis of (Poly)Phenols in Fig Peel and Whole Dried Fig through uHPLC-MS^n^

UHPLC-MS^n^ experiments were performed using an Accela UHPLC 1250 equipped with a linear ion trap-mass spectrometer (LIT-MS) (LTQ XL, Thermo Fisher Scientific Inc., Waltham, MA, USA) fitted with a heated electrospray ionization probe (H-ESI-II; Thermo Fisher Scientific Inc., Waltham, MA, USA). Separations were performed using a Blue Orchid C18 (1.8 μm particle size) column (50 × 2.1 mm (Knauer, Berlin, Germany)). The volume injected was 5 µL and the column oven was set to 40 °C. The mobile phase for gradient elution consisted of 0.1% (*v*/*v*) aqueous formic acid (solvent A) and 0.1% (*v*/*v*) formic acid in acetonitrile (solvent B). Elution was performed at a flow rate of 0.2 mL/min. The gradient started with 5% B, held until 3 min, and incremented to 40% B until 12 min, reaching 80% B three minutes later. This 80% gradient held until 16 min, followed by 5 min to re-equilibrate the column from 17 to 22 min.

Untargeted preliminary analyses were carried out in both positive and negative ionization modes. In detail, the MS worked in full scan, data-dependent MS^3^ mode (*m*/*z* range 100–1500) to investigate the anthocyanin profile of fig cultivars owing to dark-violet peel. The MS worked in ESI+ with a source voltage set to 4.5 kV, capillary voltage equal to 20 V while the tube lens voltage was 95 V. The capillary temperature was set to 275 °C with a source heater temperature equal to 300 °C. Nitrogen was used as both sheath and auxiliary gas, with values at 40 and 5 units, respectively. The Collision Induced Dissociation (CID) was equal to 15 and 35 for MS^2^ and MS^3^ experiments, respectively. An analysis in negative ESI mode was performed for all fig cultivars to investigate the profile of non-anthocyanin (poly)phenols. In detail, the MS worked in full scan, data-dependent MS^3^ mode scanning from *m*/*z* 100 to 1500. Source voltage was set to 4 kV, capillary voltage equal to −26 V, while the tube lens voltage was −78 V. The capillary temperature was set to 275 °C with a source heater temperature equal to 50 °C. Nitrogen was used as both sheath and auxiliary gas, with values at 50 and 40 units, respectively. The CID was 30 for both MS^2^ and MS^3^ experiments. Pure helium (99.9999%) was used as collision gas. Once the identification through the preliminary untargeted analyses was performed, anthocyanins were quantified in Full MS^2^ mode by selecting the specific molecular ion (M)^+^ with a CID of 35, while non-anthocyanin (poly)phenols were quantified in Full MS^2^ mode by monitoring the specific (M-H)^-^ with a CID equal to 30. Chromatograms and mass spectral data were acquired using Xcalibur software 2.1 (Thermo Fisher Scientific Inc., Waltham, MA, USA). The LC-MS characteristics of (poly)phenolic compounds are listed in Table S1. The identification was performed by comparing the MS^n^ ion spectra with the MS^n^ data stored in several online libraries such as PubChem (https://pubchem.ncbi.nlm.nih.gov/) (accessed on 16 July 2022); mzCloud (http://www.mzcloud.org/home) (accessed on 16 July 2022); Metlin (http://metlin.scripps.edu) (accessed on 16 July 2022); MoNA—Mass Bank of North America (https://mona.fiehnlab.ucdavis.edu/) (accessed on 16 July 2022) and ReSpect for Phytochemicals (http://spectra.psc.riken.jp/menta.cgi/index) (accessed on 16 July 2022). Additional MS^n^ information was obtained from previous works [10,21,31]. The quantification of fig (poly)phenols was performed by using the proper standard compound or the most structurally related compound, with details reported in Table S1.

### 3.4. Statistical Analysis

Values were reported as mean ± SE. Analysis of variance (ANOVA) was carried out through Tukey test (*p* < 0.05) using the IBM SPSS Statistics 19 software package (SPSS Inc., Chicago, IL, USA).

## 4. Conclusions

The findings obtained in the current study proved that fig peel is a valuable source of (poly)phenols, mainly rutin and 5-caffeoylquinic acid in greenish fig peel, and cyanidin-3-*O*-rutinoside in dark-violet fig peel. Thus, the consumption of unpeeled figs should be encouraged in the healthy population, following the national dietary guidance recommendation, to increase the daily intake of (poly)phenolic compounds. Moreover, since fig peel is also a plant-based by-product readily available in large amounts from the confectionary industry, its possible re-use as a (poly)phenol-rich ingredient in several food products should be considered, also within the framework of environmental impact reduction. Moreover, specifically dark-violet fig peel could be potentially used as a natural colorant.

## Figures and Tables

**Table 1:** (Poly)phenol content in fig peel.

**Compound**	**F1**	**F2**	**F3**	**F4**	**F5**	**F6**
**Phenolic acids**						
3-Caffeoylquinic acid	9.06 ± 1.51 ^b^	4.16 ± 0.40 ^c,d,e^	2.97 ± 0.11 ^d,e,f^	2.91 ± 0.44 ^d,e,f^	1.48 ± 0.58 ^f^	4.43 ± 0.56 ^c,d^
5-Caffeoylquinic acid	50.09 ± 1.06 ^d^	34.51 ± 2.34 ^d^	130.73 ± 4.77 ^a^	45.06 ± 1.05 ^d^	56.67 ± 3.18 ^c,d^	68.51 ± 5.75 ^d^
Caffeoylquinic acid isomer	22.65 ± 6.95 ^b,c,d^	7.41 ± 0.60 ^e^	32.32 ± 11.18 ^a,b^	14.95 ± 2.62 ^c,d,e^	22.75 ± 5.29 ^b,c,d^	21.60 ± 7.79 ^b,c,d,e^
**Flavan-3-ols**						
(+)-Catechin	0.44 ± 0.04 ^c^	0.55 ± 0.14 ^c^	11.48 ± 2.74 ^a^	2.84 ± 0.36 ^c^	0.93 ± 0.05 ^c^	0.32 ± 0.11 ^c^
(−)-Epicatechin	1.10 ± 0.03 ^d^	6.20 ± 0.18 ^b^	12.05 ± 0.77 ^a^	0.31 ± 0.09 ^e^	0.30 ± 0.03 ^e^	0.69 ± 0.09 ^e^
Procyanidin dimer B-type	1.74 ± 0.55 ^c,d^	1.14 ± 0.09 ^d,e^	17.14 ± 0.80 ^a^	0.23 ± 0.23 ^e^	0.33 ± 0.33 ^e^	1.05 ± 0.18 ^e^
**Flavones**						
Vitexin	0.92 ± 0.33 ^d,e^	1.20 ± 0.28 ^c,d,e^	2.05 ± 0.21 ^b,c^	1.21 ± 0.19 ^c,d,e^	0.52 ± 0.03 ^e^	1.50 ± 0.53 ^b,c,d,e^
Apigenin-*C*-hexoside	0.66 ± 0.03 ^c^	3.26 ± 0.55 ^a^	3.29 ± 0.34 ^a^	2.75 ± 0.23 ^a^	1.13 ± 0.21 ^b,c^	3.19 ± 0.72 ^a^
Luteolin-*C*-hexoside	1.18 ± 0.05 ^i^	7.57 ± 0.16 ^b^	3.16 ± 0.15 ^d^	8.91 ± 0.23 ^a^	2.21 ± 0.14 ^e,f^	5.64 ± 0.13 ^c^
Apigenin-*C*-hexoside-C-pentoside I	2.34 ± 0.27 ^c,d,e^	2.56 ± 0.37 ^c,d^	1.97 ± 0.18 ^d,e,f^	4.30 ± 0.13 ^b^	2.29 ± 0.37 ^c,d,e^	3.15 ± 0.12 ^c^
Apigenin-*C*-hexoside-C-pentoside II	0.63 ± 0.07 ^e,f^	0.87 ± 0.10 ^d,e^	0.62 ± 0.00 ^e,f^	1.72 ± 0.20 ^b^	0.94 ± 0.06 ^d^	1.41 ± 0.08 ^c^
Apigenin-*O*-rutinoside	ND	3.18 ± 0.22 ^b^	27.22 ± 1.14 ^a^	ND	ND	ND
Apigenin-*O*-rhamnoside-*C*-hexoside	1.11 ± 0.48^d^	5.71 ± 0.99 ^a,b,c^	2.99 ± 0.48 ^c,d^	6.24 ± 0.08 ^a,b^	8.31 ± 0.40 ^a^	7.61 ± 1.69 ^a,b^
Methylluteolin-*O*-rhamnoside-*C*-hexoside	1.36 ± 0.21^c^	6.23 ± 0.24 ^b^	3.06 ± 0.18 ^c^	9.06 ± 0.23 ^a^	8.43 ± 1.53 ^a,b^	6.45 ± 0.51 ^b^
**Flavonols**						
Quercetin-*O*-hexoside	45.52 ± 4.04 ^b,c^	24.86 ± 2.53 ^d^	49.75 ± 1.89 ^b^	16.79 ± 0.71 ^e,f^	11.16 ± 1.23 ^f^	26.72 ± 1.35 ^d^
Kaempferol-*O*-acetylhexoside	0.28 ± 0.10 ^b,c,d^	0.20 ± 0.03 ^d^	0.25 ± 0.05 ^c,d^	0.47 ± 0.05 ^a,b^	0.30 ± 0.07 ^b,c,d^	0.61 ± 0.06 ^a^
Quercetin-*O*-acetylhexoside I	5.89 ± 0.13 ^b,c^	4.13 ± 0.19 ^d,e^	5.13 ± 0.44 ^c,d^	4.34 ± 0.03 ^d,e^	3.74 ± 0.61 ^e,f^	3.74 ± 0.46 ^e,f^
Quercetin-*O*-acetylhexoside II	1.21 ± 0.26 ^e,f^	1.66 ± 0.22 ^d,e,f^	2.15 ± 0.11 ^c,d^	1.98 ± 0.15 ^c,d,e^	1.12 ± 0.14 ^d^	1.59 ± 0.18 ^d,e,f^
Kaempferol-*O*-rutinoside	6.90 ± 0.25 ^e^	8.89 ± 1.59 ^c,d,e^	10.08 ± 1.11 ^b,c,d^	11.07 ± 1.00 ^b,c^	7.39 ± 0.37 ^d,e^	10.03 ± 1.18 ^b,c,d^
Quercetin-3-*O*-rutinoside	119.46 ± 17.56 ^b,c,d^	101.27 ± 10.06 ^d,e^	110.20 ± 8.04 ^c,d^	134.33 ± 10.35 ^b,c^	100.47 ± 3.39 ^c,d^	113.29 ± 17.84 ^c,d^
**Anthocyanins**						
Cyanidin-3-*O*-glucoside	ND	61.47 ± 4.43 ^b^	208.92 ± 4.62 ^a^	ND	ND	ND
Cyanidin-*O*-malonyl-glucoside	ND	7.18 ± 0.49 ^b^	24.21 ± 1.29 ^a^	ND	ND	ND
Cyanidin-3-*O*-rutinoside	ND	643.36 ± 27.49 ^b^	2610.20 ± 100.67 ^a^	ND	ND	ND
Cyanidin-*O*-dihexoside	ND	9.68 ± 0.38 ^b^	25.10 ± 0.29 ^a^	ND	ND	ND
(Epi)catechin-cyanidin-*O*-rutinoside	ND	4.17 ± 0.12 ^b^	14.39 ± 0.93 ^a^	ND	ND	ND
Cyanidin-*O*-rutinoside dimer I	ND	0.60 ± 0.09 ^c^	1.68 ± 0.16 ^a^	ND	ND	ND
Cyanidin-*O*-rutinoside dimer II	ND	0.74 ± 0.09 ^b^	2.47 ± 0.19 ^a^	ND	ND	ND
Cyanidin-*O*-rutinoside dimer III	ND	0.13 ± 0.03	ND	ND	ND	ND
**Compound**	**F7**	**F8**	**F9**	**F10**	**F11**	**F12**
**Phenolic acids**						
3-Caffeoylquinic acid	9.14 ± 0.67 ^b^	16.86 ± 1.75 ^a^	5.76 ± 0.53 ^c^	1.14 ± 0.15 ^f^	2.14 ± 0.28 ^d,e,f^	1.88 ± 0.21 ^e,f^
5-Caffeoylquinic acid	64.93 ± 3.38 ^c,d^	110.60 ± 17.12 ^a,b^	96.75 ± 2.14 ^b^	39.43 ± 4.94 ^d^	39.28 ± 4.38 ^d^	82.04 ± 1.75 ^b,c^
Caffeoylquinic acid isomer	42.69 ± 6.31 ^a^	25.67 ± 1.92 ^b,c^	17.94 ± 1.00 ^b,c,d,e^	9.88 ± 1.43 ^d,e^	10.07 ± 1.58 ^d,e^	19.06 ± 1.25 ^b,c,d,e^
**Flavan-3-ols**						
(+)-Catechin	0.21 ± 0.06 ^c^	0.24 ± 0.21 ^c^	1.91 ± 0.09 ^c^	0.43 ± 0.02 ^c^	1.48 ± 0.58 ^c^	7.70 ± 1.99 ^b^
(−)-Epicatechin	0.66 ± 0.06 ^e^	3.10 ± 0.40 ^c^	3.87 ± 0.03 ^c^	0.28 ± 0.06 ^e^	0.25 ± 0.03 ^e^	0.66 ± 0.15 ^d,e^
Procyanidin dimer B-type	ND	0.50 ± 0.04 ^e^	4.08 ± 0.45 ^b^	ND	0.52 ± 0.20 ^e^	2.79 ± 0.23 ^c^
**Flavones**						
Vitexin	0.52 ± 0.06 ^e^	2.36 ± 0.77 ^a,b^	1.85 ± 0.30 ^b,c,d^	3.20 ± 0.46 ^a^	1.14 ± 0.13 ^c,d,e^	0.55 ± 0.06 ^e^
Apigenin-*C*-hexoside	1.25 ± 0.21 ^b,c^	0.35 ± 0.06 ^c^	1.59 ± 0.12 ^b^	3.40 ± 0.25 ^a^	1.08 ± 0.11 ^b,c^	0.36 ± 0.04 ^c^
Luteolin-*C*-hexoside	1.79 ± 0.19 ^f,g,h^	2.57 ± 0.29 ^e^	1.99 ± 0.09 ^f,g^	5.81 ± 0.16 ^c^	1.41 ± 0.07 ^h,i^	1.68 ± 0.17 ^g,h^
Apigenin-*C*-hexoside-C-pentoside I	4.14 ± 0.80 ^b^	1.19 ± 0.06 ^f^	5.43 ± 0.34 ^a^	1.53 ± 0.15 ^e,f^	2.36 ± 0.22 ^c,d,e^	4.22 ± 0.23 ^b^
Apigenin-*C*-hexoside-C-pentoside II	2.05 ± 0.15 ^a^	0.21 ± 0.05 ^g^	1.45 ± 0.15 ^b,c^	0.24 ± 0.04 ^g^	0.53 ± 0.03 ^f^	1.05 ± 0.03 ^d^
Apigenin-*O*-rutinoside	ND	0.93 ± 0.11 ^c^	ND	ND	ND	ND
Apigenin-*O*-rhamnoside-*C*-hexoside	6.34 ± 1.22 ^a,b^	0.80 ± 0.48 ^d^	5.16 ± 1.54 ^b,c^	1.68 ± 0.25 ^d^	8.21 ± 2.16 ^a,b^	0.36 ± 0.03 ^d^
Methylluteolin-*O*-rhamnoside-*C*-hexoside	6.40 ± 0.73 ^b^	1.52 ± 0.26 ^c^	7.10 ± 0.99 ^a,b^	1.97 ± 0.34 ^c^	6.75 ± 1.50 ^b^	1.16 ± 0.05 ^c^
**Flavonols**						
Quercetin-*O*-hexoside	21.51 ± 1.05 ^d,e^	56.96 ± 3.82 ^a^	43.47 ± 2.22 ^c^	12.91 ± 1.40 ^f^	14.92 ± 1.04 ^f^	25.31 ± 0.71 ^d^
Kaempferol-*O*-acetylhexoside	0.45 ± 0.07 ^a,b^	0.42 ± 0.06 ^b,c^	0.33 ± 0.11 ^b,c,d^	0.20 ± 0.05 ^d^	0.23 ± 0.01 ^c,d^	0.20 ± 0.04 ^d^
Quercetin-*O*-acetylhexoside I	5.62 ± 0.30 ^c^	7.85 ± 0.30 ^a^	6.93 ± 0.21 ^a,b^	2.99 ± 0.47 ^f^	3.62 ± 0.42 ^e,f^	6.79 ± 0.34 ^a,b^
Quercetin-*O*-acetylhexoside II	2.64 ± 0.16 ^c^	4.90 ± 0.71 ^a^	3.66 ± 0.20 ^b^	0.97 ± 0.06 ^f^	1.38 ± 0.13 ^d,e,f^	3.57 ± 0.18 ^b^
Kaempferol-*O*-rutinoside	3.04 ± 0.11 ^f^	18.81 ± 2.30 ^a^	12.72 ± 0.25 ^b^	2.58 ± 0.37 ^f^	2.57 ± 0.52 ^f^	10.98 ± 0.62 ^b,c^
Quercetin-3-*O*-rutinoside	73.50 ± 2.52 ^e^	197.58 ± 18.17 ^a^	152.16 ± 10.96 ^b^	74.55 ± 8.55 ^e^	87.52 ± 3.64 ^d,e^	135.22 ± 6.68 ^b,c^
**Anthocyanins**						
Cyanidin-3-*O*-glucoside	ND	14.01 ± 0.60 ^c^	17.36 ± 0.93 ^c^	ND	ND	ND
Cyanidin-*O*-malonyl-glucoside	ND	1.89 ± 0.18 ^c^	1.18 ± 0.18 ^c^	ND	ND	ND
Cyanidin-3-*O*-rutinoside	ND	222.48 ± 9.19 ^c^	115.91 ± 6.23 ^d^	ND	ND	ND
Cyanidin-*O*-dihexoside	ND	4.16 ± 0.05 ^d^	7.83 ± 0.63 ^c^	ND	ND	ND
(Epi)catechin-cyanidin-*O*-rutinoside	ND	1.17 ± 0.01 ^c^	1.16 ± 0.07 ^c^	ND	ND	ND
Cyanidin-*O*-rutinoside dimer I	ND	1.17 ± 0.01 ^b^	ND	ND	ND	ND
Cyanidin-*O*-rutinoside dimer II	ND	0.12 ± 0.01 ^c^	ND	ND	ND	ND
Cyanidin-*O*-rutinoside dimer III	ND	ND	ND	ND	ND	ND

Data expressed as mean values of μg/g (dry weight) with their standard errors (*n* = 3). F1: Bianco; F2: Brogiotto nero; F3: Corbo; F4: Brogiotto bianco; F5: Paradiso; F6: Verdino; F7: Pecciolo bianco; F8: Portogallo; F9: Pecciolo nero; F10: Dottato; F11: Gigante di Carmignano; F12: Perticone. ND: not detected. Different letter indicates significantly different values (*p* < 0.05).

**Table 2:** Occurrence of minor (poly)phenols in fig peel.

Compound	F1	F2	F3	F4	F5	F6	F7	F8	F9	F10	F11	F12
**Phenolic acids**												
Vanillic acid derivative I	+	−	−	−	+	+	+	+	+	+	+	+
Vanillic acid derivative II	+	+	+	+	+	+	+	+	+	+	+	+
Dihydroxybenzoic acid-*O*-pentoside	+	+	+	+	+	+	+	+	+	+	+	+
Hydroxybenzoic acid-*O*-hexoside	+	+	+	+	+	+	+	+	+	+	+	+
Dihydroxybenzoic acid-*O*-hexoside	+	+	+	+	+	+	+	+	+	+	+	+
Caffeic acid-*O*-hexoside	−	+	+	−	−	−	−	−	−	−	−	−
Homovanillic acid-*O*-hexoside	+	+	+	+	+	+	+	+	+	+	+	+
Ferulic acid-*O*-hexoside	+	+	+	+	+	+	+	+	+	+	+	+
Sinapic acid-*O*-hexoside	+	+	+	+	+	+	+	+	+	+	+	+
**Flavanones**												
Pinocembrin	−	+	+	−	−	+	+	+	+	+	+	+
Naringenin	−	+	+	+	+	+	−	+	+	+	+	+
Naringenin-like	+	+	+	+	+	+	−	+	−	−	+	+
Naringenin-*O*-hexoside	−	+	+	−	−	+	+	+	+	+	+	+
**Flavones**												
Apigenin	+	+	+	+	+	+	+	+	+	+	+	+
Luteolin-*C*-hexoside-*O*-rhamnoside	+	+	+	+	+	+	+	+	+	+	+	+
**Flavonols**												
Taxifolin-*O*-hexoside	+	+	+	−	+	+	−	+	+	+	+	+

**Table 3:** Fig cultivars investigated in the present study.

Sample Code	Cultivar	Peel Color
F1	Bianco	Light green
F2	Brogiotto nero	Dark-violet
F3	Corbo	Dark-violet
F4	Brogiotto bianco	Light green
F5	Paradiso	Green
F6	Verdino	Bright green
F7	Pecciolo bianco	Light green
F8	Portogallo	Dark-violet
F9	Pecciolo nero	Dark-violet
F10	Dottato	Light green
F11	Gigante di Carmignano	Green
F12	Perticone	Green

## Data Availability

Data are contained within the article and in supplementary material.

## Supplementary Materials

The following are available online at https://www.mdpi.com/article/10.3390/plants11223073/s1, Table S1: Chromatographic and mass spectrometric characteristics of (poly)phenols identified in fig peel and whole dried fig; Figure S1: Chromatographic profiles of caffeoylquinic acids and their MS/MS spectra; Figure S2: Chromatographic profile of quercetin-3-*O*-rutinoside and its MS/MS spectra; Figure S3: Chromatographic profiles of cyanidin-3-*O*-glucoside and cyanidin-3-*O*-rutinoside with their MS/MS spectra.
