# Supplementary material for: Characterization of the (Poly)Phenolic Fraction of Fig Peel: Comparison among Twelve Cultivars Harvested in Tuscany
Source: Plants (Basel). 2022 Nov 13;11(22):3073. doi: 10.3390/plants11223073 (PMC9697167; doi:10.3390/plants11223073)

Characterization of the (Poly)Phenolic Fraction of Fig Peel: Comparison among Twelve Cultivars Harvested in Tuscany

Luca Calani [1], Letizia Bresciani [1,*], Margherita Rodolfi [1], Daniele Del Rio [1], Raffaella Petruccelli [2], Cecilia Faraloni [2] and Tommaso Ganino [1,2,*]

[1] Department of Food and Drug, University of Parma, Parco Area delle Scienze 27/A, 43124 Parma, Italy
[2] Institute of BioEconomy (IBE-CNR), via Madonna del Piano 10, 50019 Sesto Fiorentino (FI), Italy
* Correspondence: letizia.bresciani@unipr.it (L.B.); tommaso.ganino@unipr.it (T.G.); Tel.: +39-0521-903906 (L.B.); +39-0521-905597 (T.G.)

Supplementary Material

Table S1. Chromatographic and mass spectrometric characteristics of (poly)phenols identified in fig peels and whole dried fig.

Compound	RT (min)	[M-H]⁻ (*m/z*)	MS² ions (*m/z*)	MS³ ions (*m/z*)	Standard compound used for quantification
**Phenolic acids**					
Vanillic acid derivative I	7.46	167	**152**,167		
Vanillic acid derivative II	8.96	167	**123**,152		
Dihydroxybenzoic acid-*O*-pentoside	3.21	285	**153**,152,165,151,163,225,109	(153)109,135	
Hydroxybenzoic acid-*O*-hexoside	4.07	299	**137**	(137)93	
Dihydroxybenzoic acid-*O*-hexoside	1.75	315	**153**,152,165,109	(153)109,153,108	
Caffeic acid-*O*-hexoside	3.86	341	**179**,297,113,143,173		
Homovanillic acid-*O*-hexoside	2.97	343	**181**,137,163		
3-Caffeoylquinic acid	2.70	353	**191**,179	(191)127,173,85,93	3-Caffeoylquinic acid
5-Caffeoylquinic acid	6.24	353	**191**,179	(191)127,173,85,93	5-Caffeoylquinic acid
Caffeoylquinic acid isomer	7.27	353	**191**,179	(191)127,173,85,93	5-Caffeoylquinic acid
Ferulic acid-*O*-hexoside**	6.14	401	**355**,193	(355)193	
Sinapic acid-*O*-hexoside**	6.93	431	**385**,223,269	(385)223	
**Flavan-3-ols**					
(+)-Catechin	4.25	289	**245**,205,179,231		(+)-Catechin
(-)-Epicatechin	7.19	289	**245**,205,179,231		(+)-Catechin
Procyanidin dimer B-type	3.57	577	**425**,451,407,289,287,467		Procyanidin B2
**Flavanones**					
Pinocembrin	12.39	255	**213**,211,151,187		
Naringenin	12.17	271	**151**,177		
Naringenin-like	12.33	271	**151**,177		
Naringenin-*O*-hexoside	10.74	433	**271**,295	(271)151,177,107	
**Flavones**					
Apigenin	12.51	269	**269**,225,149,201,151,226,227	(269)269,225,149,181	
Vitexin	8.88	431	**311**,341		Vitexin
Apigenin-*C*-hexoside	9.07	431	**311**,341,413		Vitexin
Luteolin-*C*-hexoside	8.58	447	**327**,357,429,285	(327)299,284,327,283	Vitexin
Apigenin-*C*-hexoside-*C*-pentoside I	8.46	563	**443**,473,545,503,353,383		Vitexin
Apigenin-*C*-hexoside-*C*-pentoside II	8.51	563	**443**,473,545,503,353,383		Vitexin
Apigenin-*O*-rutinoside	7.96	577	**269**,283,225,311		Rutin
Apigenin-*O*-rhamnoside-*C*-hexoside	9.02	577	**413**,293,457,341		Vitexin

Luteolin-*C*-hexoside-*O*-rhamnoside	8.54	593	**473**,429,285,357,339,309,284,327		Vitexin
Methylluteolin-*O*-rhamnoside-*C*-hexoside	9.21	607	**443**,323,353,487		Vitexin
**Flavonols**					
Quercetin-*O*-hexoside	9.28	463	**301**,300,302	(301)179,151,273,257,283	Rutin
Taxifolin-*O*-hexoside	5.69	465	**303**,285	(303)285,177,125,259	
Kaempferol-*O*-acetylhexoside	10.22	489	**285**,327	(285)257,267,241,229,163,199,213	Rutin
Quercetin-*O*-acetylhexoside I	9.40	505	**301**,300,463	(301)179,151,257,229	Rutin
Quercetin-*O*-acetylhexoside II	9.58	505	**301**,300,463	(301)179,151,257,229	Rutin
Kaempferol-*O*-rutinoside	9.66	593	**285**,286,257	(285)257,267,229,241,213	Rutin
Quercetin-3-*O*-rutinoside	9.1	609	**301**,299,343	(301)179,151,257,273	Rutin
**Anthocyanins**					
Cyanidin-3-*O*-glucoside	6.95	449*	**287**,286	(287)287,213,231,241,259,189	Cyanidin-3-*O*-glucoside
Cyanidin-*O*-malonylglucoside	8.86	535*	**287**,535,491,449	(287)287,213,259,241,231,269,189	Cyanidin-3-*O*-glucoside
Cyanidin-3-*O*-rutinoside	7.41	595*	**595**,287,449	(595)287,449	Cyanidin-3-*O*-rutinoside
Cyanidin-*O*-dihexoside	5.57	611*	**611**	(611)449,287	Cyanidin-3-*O*-glucoside
(epi)catechin-cyanidin-rutinoside	6.33	883*	**883**	(883)575,423,737,329,557	Cyanidin-3-*O*-rutinoside
Cyanidin-*O*-rutinoside dimer I	6.63	1189*	**1189**	(1189)881,1043,735,573	Cyanidin-3-*O*-rutinoside
Cyanidin-*O*-rutinoside dimer II	7.57	1189*	**1189**	(1189)881,1043,735,573	Cyanidin-3-*O*-rutinoside
Cyanidin-*O*-rutinoside dimer III	8.34	1189*	**1189**	(1189)881,1043,735,573	Cyanidin-3-*O*-rutinoside

*Anthocyanins analysed in ESI+ $[M]^+$. ** Formate adduct. Product ions are listed in their order of relative ionic abundance and the highest fragment ion is reported in bold type.

Figure S1. Chromatographic profiles of caffeoylquinic acids and their MS/MS spectra.

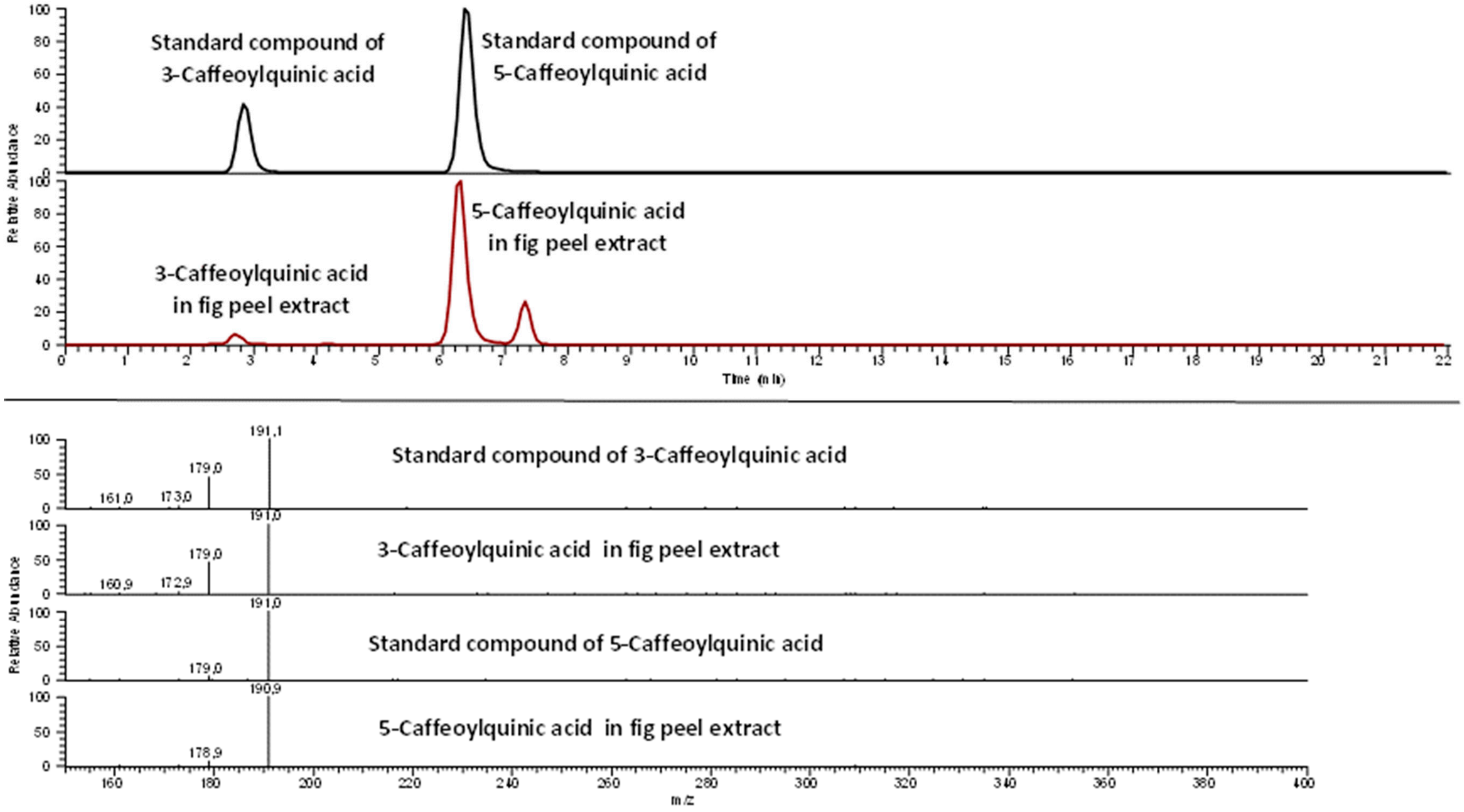

Figure S2. Chromatographic profile of quercetin-3-O-rutinoside and its MS/MS spectra.

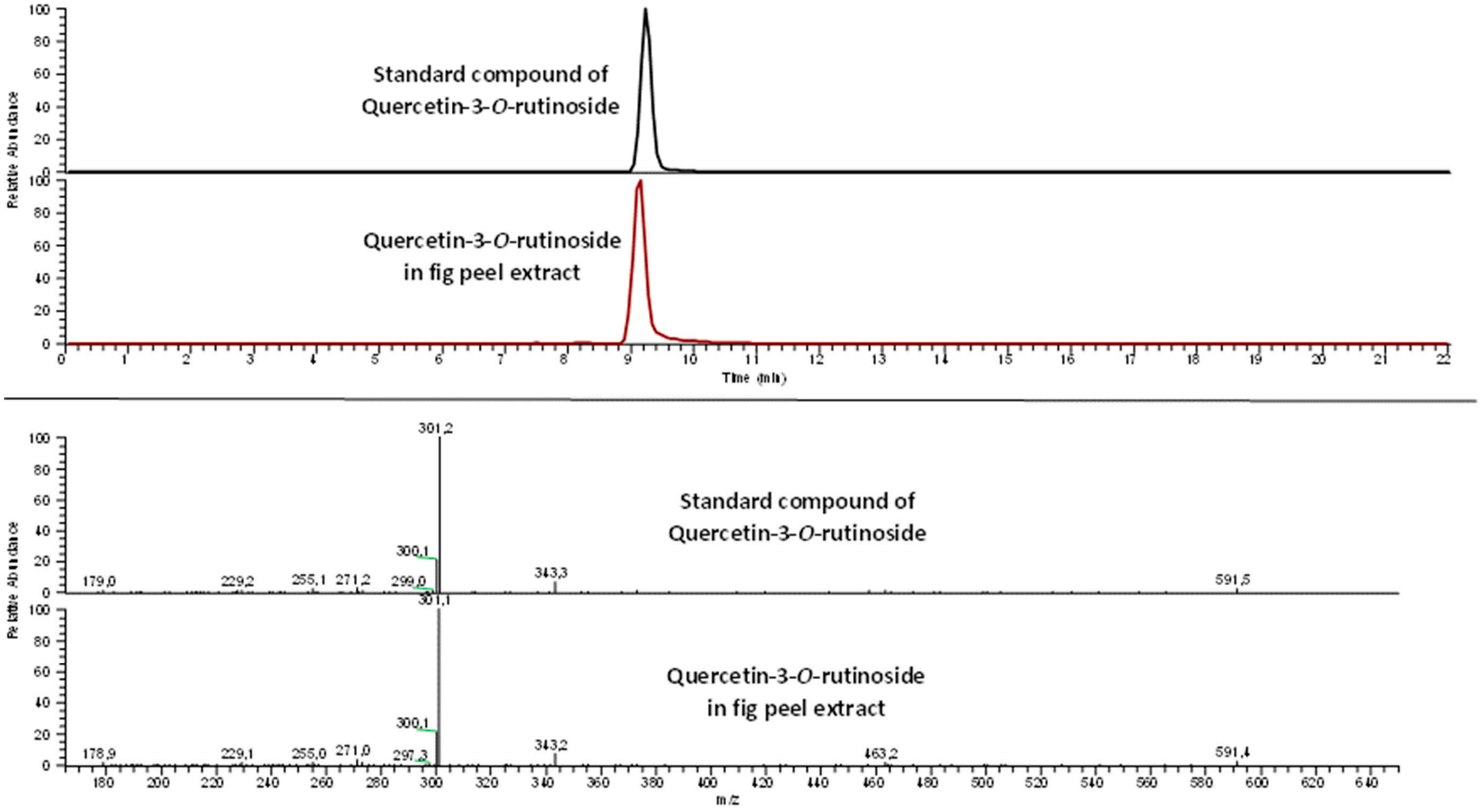

Figure S3. Chromatographic profiles of cyanidin-3-*O*-glucoside and cyanidin-3-*O*-rutinoside with their MS/MS spectra.

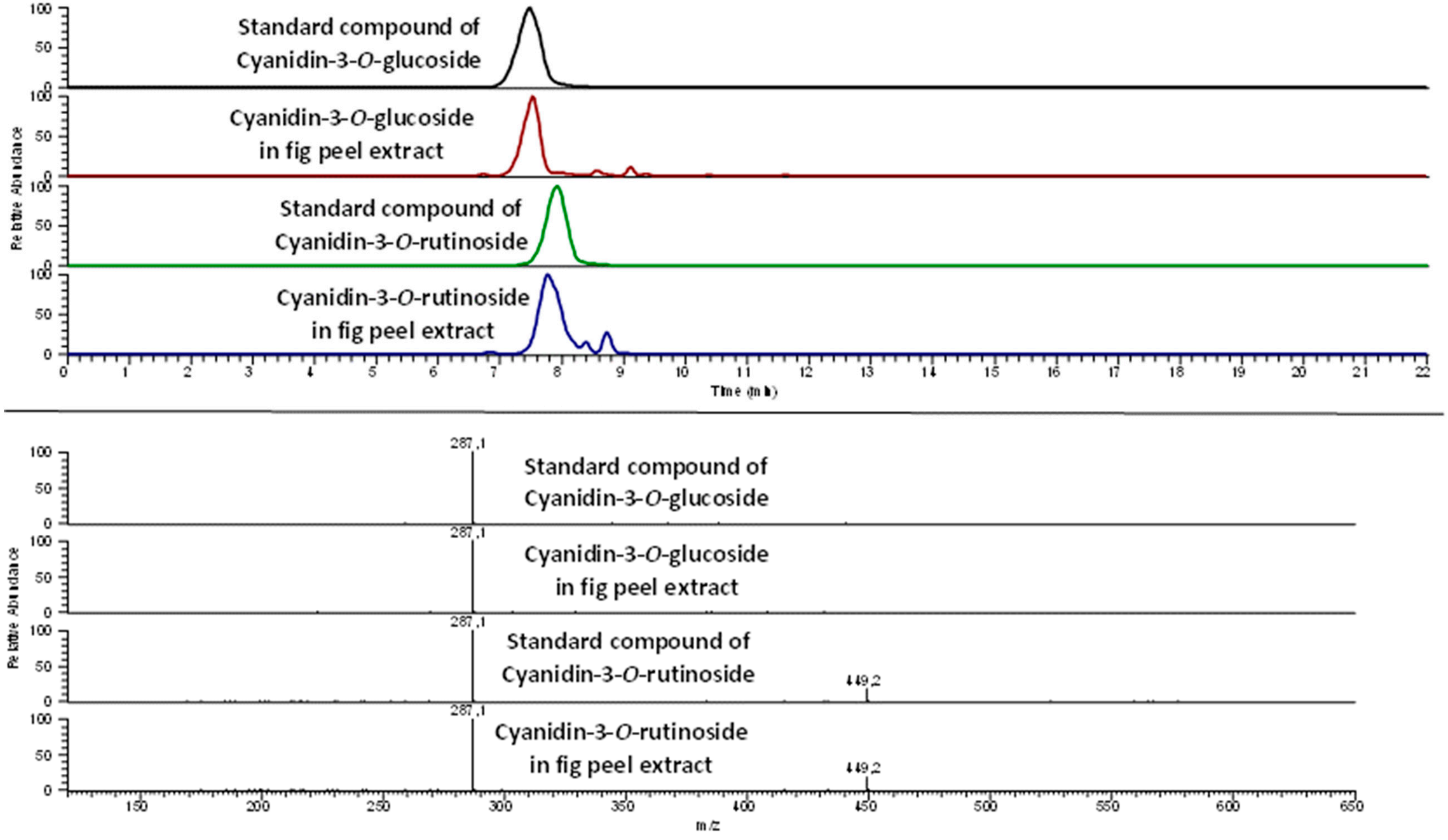

Supplement: Supplementary file 1 [file plants-11-03073-s001.zip › plants-2014573-supplementary.pdf]
